# Resilience-Building for Mental Health among Early Childhood Educators: A Systematic Review and Pilot-Study towards an EEG-VR Resilience Building Intervention

**DOI:** 10.3390/ijerph19074413

**Published:** 2022-04-06

**Authors:** Rumaisa Abu Hasan, Muhamad Saiful Bahri Yusoff, Tong Boon Tang, Yasir Hafeez, Mazlina Che Mustafa, Masayu Dzainudin, Juppri Bacotang, Ubaid M. Al-Saggaf, Syed Saad Azhar Ali

**Affiliations:** 1 Centre for Intelligent Signal and Imaging Research (CISIR), Electrical and Electronics Engineering Department, University Teknologi PETRONAS, Seri Iskandar 32610, Perak, Malaysia; rumaisa_19000937@utp.edu.my (R.A.H.); tongboon.tang@utp.edu.my (T.B.T.); yasir_hafeez@ymail.com (Y.H.); 2 Department of Medical Education, School of Medical Sciences, University Sains Malaysia, Kota Bharu 16150, Kelantan, Malaysia; msaiful_bahri@usm.my; 3 National Child Development Research Centre, University Pendidikan Sultan Idris, Tanjong Malim 35900, Perak, Malaysia; mazlina.cm@fpm.upsi.edu.my (M.C.M.); masayu@fpm.upsi.edu.my (M.D.); 4 Faculty of Psychology and Education, University Malaysia Sabah, Kota Kinabalu 88400, Sabah, Malaysia; juppri@ums.edu.my; 5 Center of Excellence in Intelligent Engineering Systems (CEIES), King Abdulaziz University, Jeddah 21589, Saudi Arabia; usaggaf@kau.edu.sa; 6 Department of Electrical and Computer Engineering, King Abdulaziz University, Jeddah 21589, Saudi Arabia

**Keywords:** resilience, work stress, intervention, early childhood education, EEG, virtual reality

## Abstract

Resilience is a key factor that reflects a teacher’s ability to utilize their emotional resources and working skills to provide high-quality teaching to children. Resilience-building interventions aim to promote positive psychological functioning and well-being. However, there is lack of evidence on whether these interventions improve the well-being or mental health of teachers in early childhood education (ECE) settings. This review examined the overall effectiveness of resilience-building interventions conducted on teachers working in the ECE field. A systematic approach is used to identify relevant studies that focus on resilience-building in countering work stress among early childhood educators. Findings from this review observed a preference of group approaches and varying durations of interventions. This review highlights the challenges of the group approach which can lead to lengthy interventions and attrition amongst participants. In addition to the concerns regarding response bias from self-report questionnaires, there is also a lack of physiological measures used to evaluate effects on mental health. The large efforts by 11 studies to integrate multiple centres into their intervention and the centre-based assessment performed by four studies highlight the need for a centre-focused approach to build resilience among teachers from various ECE centres. A pilot study is conducted to evaluate the feasibility of an integrated electroencephalography–virtual reality (EEG-VR) approach in building resilience in teachers, where the frontal brain activity can be monitored during a virtual classroom task. Overall, the findings of this review propose the integration of physiological measures to monitor changes in mental health throughout the resilience-building intervention and the use of VR as a tool to design a unique virtual environment.

## 1. Introduction

The increase in work-related stress among adults is a raising concern throughout the world. In 2002, the World Health Organization published a monograph describing the alarming mental health problem as one of the leading causes of disability and disease world-wide [1]. This problem has been recognized as detrimental in both poor and rich countries, and persists in the population regardless of age, gender, and social strata [2,3,4,5]. Among working adults, teachers are often reported to face high levels of stress in their working field [4,6,7,8,9,10], highlighting how emotionally challenging teaching is as an occupation. Teachers of young children are at risk of mental health complications, with literature reporting that 6–36% of early childhood educators meet the clinical cut-off for depressive symptoms [11]. In a large-scale study on 674 childcare workers, 36% of the cohort assessed had clinically depressive symptoms [12]. A study involving teachers from 73 Head Start programs reported 25% of the teachers had clinically depressive symptoms [13]. These statistics raise concern on the well-being of early childhood educators, both in terms of their emotional and physical health [14,15,16,17], and the consequences on children’s development [13,18,19].

### 1.1. Challenges of the Workload on Mental Health of Teachers

Teachers in early childhood education (ECE) hold a crucial role in building social–emotional and social–cognitive competence in children [11]. With the reform of policymakers to encourage better and broader education, heavy responsibilities are placed upon early childhood educators in addressing goals in aspects such as teaching and learning, teacher–child ratio, curriculum, and operation schedule [20]. Rapid changes in the approaches and expectations placed upon ECE increase the level of work stress faced by adults within this professional field. A study conducted in a rural area revealed the level of work stress among early childhood educators increased with higher classroom workload and poor availability of technology resources for teaching [21]. The level of work stress can also be reflected in a qualitative study, where respondents highlighted various responsibilities such as the need to teach many languages to children, holistic approaches in the curriculum, and monitoring and supervision on the quality of teaching, all of which contribute to the increased workload of educators [22].

Despite the strong relationship between teaching quality and children’s development in ECE, little attention has been given to the well-being of teachers [14,23,24]. Early childhood educators are faced with stress inducers such as heavy workload, time constraints, lack of intellectual stimulation, inter-personal relationships with colleagues and parents and teacher–children relationships, and in some locations, poor working conditions [19]. Often enough, the job scope of early childhood educators overlaps with child-care responsibilities, which the public perceives as a lesser occupation [25]. Those working in childcare have been observed to exhibit poor health behaviour and status (e.g., high obesity prevalence, smoking, drinking sweet beverages) [12]. Constant exposure to a high-stress environment without effective coping mechanisms regarding mental stress can lead to emotional and physical exhaustion, depersonalization, and decreased job performance and commitment, followed by depression and other detrimental clinical health problems [19].

The increasing complexity and public expectation of quality childhood education observed in related studies, for example the authors in [21,22,26] suggest a need to address work stress faced by these professionals. In one review, Hall-Kenyon et al. (2014) highlighted that literature on early childhood educators measured well-being based on external influences on one’s feeling rather than internal factors linked to self-belief [14]. The review also emphasized the need to address well-being of the early childhood educators in terms of both personal and professional concerns. Literature is advocating towards finding effective approaches to coping with stress for teachers to support themselves in their mental health along with honing teaching skills relevant to managing early education environments [8,27,28]. A recent updated review on the well-being of early childhood educators highlighted the need for sufficient information to standardize the measurement of well-being among early childhood educators [23,29].

### 1.2. Role of Resilience in Supporting Early Childhood Teachers

Regarding the stressful working environment faced by those in teaching professions, researchers have explored key factors that contribute to teachers remaining in the profession, and how they utilize their resources to develop professionally and build resilience to provide high-quality teaching despite the daily adversities [30,31]. In the face of prolonged periods of adversity, resilience is the outcome of a dynamic process that promotes and maintains successful recovery of stable mental health [32]. Resilient teachers cultivate their problem-solving skills and maintain a work–life balance. They can recognize their own stress level and take action to remedy the situation through social or physical activities [31]. In addition to one’s emotional resources, the knowledge of effective coping strategies facilitates in strengthening the teacher’s resilience [30]. These strategies are dependent on the individual and the leadership and management of the ECE centre [31,33].

### 1.3. Rationale and Objective of Review

Reforms made by policymakers to improve children’s education often emphasize teaching quality, but less attention is given to the well-being and mental health of early childhood educators. Literature has shown a significant link between the well-being of teachers and the development of children. Increased interest in resilience has been hypothesized to promote positive psychological functioning and well-being [34]. Resilience-building intervention aims to equip individuals with resources and skills to prevent the negative effects of future exposure to stressors [35]. However, there is unclear evidence on the efficacy of these interventions and their effect on resilience among early childhood educators. Hence, the objective of this systematic review is to discuss the overall implementation of resilience-building interventions in terms of program approach, duration and measuring instruments used to build resilience in teachers working in an ECE setting. Moreover, we also present preliminary findings of teachers’ frontal brain activity during a virtual reality (VR) classroom as a proposed approach towards building resilience using physiological measures.

## 2. Methodology

The aim of this review is to discuss the overall implementation of resilience-building interventions conducted among teachers working in an ECE setting. To identify relevant studies, this systematic review followed the Preferred Reporting Items for Systematic Reviews and Meta-Analyses (PRISMA) protocol.

### 2.1. Search Strategy and Eligibility Criteria

The host databases PubMed, Scopus, Web of Science and Google Scholar were used to perform keyword searches for articles, up to and including December 2021. The keywords used to search for articles were: (resilience OR resiliency) AND (work stress) AND (teacher OR educator) AND (intervention OR training OR program OR programme OR module) AND (preschool OR early childhood OR kindergarten). In the identification phase, the keywords were screened in the title and abstract of each article, without restriction on publication date. The articles were screened for research related to resilience interventions conducted in ECE settings. In this review, we define the ECE as education and care-taking of infants to 6-year-old children. Below are the eligibility criteria used to include articles obtained from the database search:

1. The study must include training or interventions to develop resilience or psychosocial well-being of professionals in an ECE setting to withstand or recover from stress or adversity faced at work;
2. The study must involve data collection involving in-service teachers working in ECE settings;
3. The study must evaluate the outcome of the intervention based on changes in well-being, mental health, or performance outcomes of participants;
4. The study is originally written in the English language.

### 2.2. Study Selection

**Figure 1:**
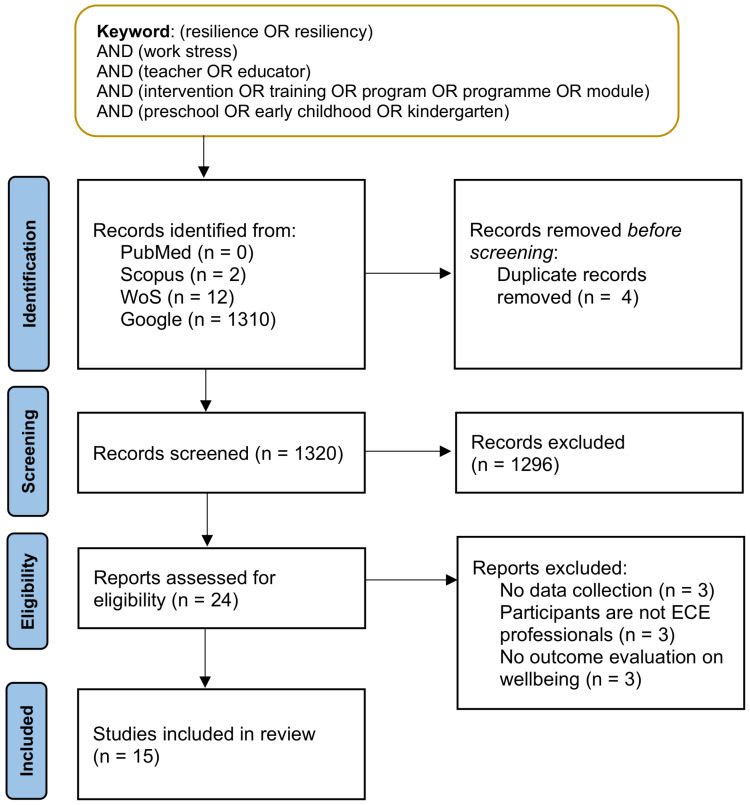
Flow diagram of study selection following PRISMA protocol. Note: ECE—early childhood education.

The search identified 1324 papers and 4 were removed as duplicates. Papers were screened by the abstract using the eligibility criteria to determine the inclusion of paper. Another 1296 papers were removed for reasons including not being relevant, having no intervention involved, participants not being professionals working in ECE settings or the article not being written in the English language. Twenty-four full-text papers were retrieved related to interventions implemented in an ECE setting to build teacher’s resilience towards work stress. Nine papers were removed as the papers focused on introducing a proposed resilience-building intervention for early childhood educators but with no data collection reported. Hence, a total of 15 papers were reviewed, and 9 are academic theses and 6 are published journal articles. The flow of study selection is shown in Figure 1.

## 3. Results

This section presents the overview of selected studies included based on the PRISMA protocol. Following the overview, studies were described based on the duration of interventions, implementation of a needs assessment prior to the intervention, characteristics of recruited participants, and the instruments used to measure resilience, well-being, or work stress in teachers. These criteria are used to discuss the preferences of approaches and instruments, and the challenges in conducting resilience-building interventions for ECE professionals, in Section 4.

### 3.1. Overview of Interventions in Selected Studies

A total of 15 articles were reviewed in this systematic review. Table 1 provides an overview of each intervention, listed according to the year of implementation. Five of the interventions employed a group-focused approach, where activities were conducted in a group of participants, and four studies adopted a mixed-approach for their intervention. Meanwhile, in the six interventions where participants completed the activities individually, three of the interventions provided self-paced modules and one intervention involved a biofeedback approach towards building resilience among teachers.

Beginning with the earliest study by Bokaba (2011), the Personal Growth Programme was developed to provide emotional support to teachers as observed from the needs assessment conducted during the early stage of research [36]. The first session of the intervention was self-exploration, where participants created a tree of life collage and focused on the positive experiences in their life; the second session was self-reflection, where the participant role-played as a child to understand how children perceive the world; the third session named “circle of friends” aimed to set up a networking group with the plan to provide support for one another. These activities were chosen as a channel for the participants to explore and apply their self-knowledge and emotions into their life experiences [36].

One study took a unique approach by assessing the efficacy of implementing an exercise program specifically for employees during their lunch break [37]. Prior to interviewing managers in selected preschools, an online survey conducted by Júníusdóttir (2015) to assess the prevalence of preschools with an employee-integrated exercise program revealed only eight preschools in Iceland integrated such programs. The drive for implementation stemmed from the concern for physical and mental health of employees. Healthier employees have higher job satisfaction and lower absenteeism. In addition, this helps in them becoming role models for children. Two preschools were recruited where the employees were given a minimum of 20–30 min during their breaks to participate in outdoor exercise activities that mainly involved running, jogging, and walking in groups. Each group was allowed to join the exercise activities twice a week, with one preschool running the sessions on Tuesdays and Thursdays. The exercise program received a high participation rate in both preschools.

Another approach that has long been integrated into early childhood development is reflective supervision. Reflective supervision uses the concept of reflection whilst in action as an approach to problem solving [38]. Lepore (2015) recruited five centres that provided reflective supervision interventions for their teachers. Through reflective supervision, one reflective supervisor works with the same group of teachers throughout the school year. The program followed contents covered in the manual “Reflective Supervision and Leadership in Infant and Early Childhood Programs”. In addition to the reflective supervision meetings, each centre provided different Early Childhood Mental Health Consultation (ECMHC) services to their teachers. The ECMHC is a collaborative approach between mental health and ECE professionals to enhance mental health among children from birth to age six. In the five centres recruited by Lepore (2015), the ECMHC supports provided for the teacher varied in terms of activities and additional hours.

Christian (2017) recruited 67 kindergarten-to-fifth-grade teachers from six elementary schools to participate in a free web-based training program [39]. The participants were divided into control and intervention groups, where the control group attended meetings as a professional learning community to discuss social and emotional functioning of teachers, share their resources and discuss practices to improve students’ classroom behaviour. The intervention group attended lessons taken from the ACHIEVER Adult Resilience Curriculum (ARC) via a massive open online course. The curriculum is a wellness-promotion training program designed to assist teachers in becoming resilient by learning and applying specific practices that contribute to reducing stress and promoting positive outcomes. The meetings for both groups were conducted for approximately 2 h in 8 weekly meetings [39].

Similar to [36], Nilsson et al. (2017) also took a centre-focused approach in developing an intervention with a salutogenic perspective to address challenges faced by early childhood educators [40]. Topics identified from the needs assessment as important for the well-being of teachers were used as the starting point for collegial reflection during the intervention. The flow of discussion throughout the meetings followed a three-phase wheel of reflection, beginning with the description of a case, followed with the evaluation of both positive and negative aspects, and finally, the conclusion of what could have been done differently. The 21 participants recruited were teachers from preschool to sixth grade classes. They were divided into groups based on the grades they taught: two groups were teachers for preschool-to-third-grade classes and one group were teachers for 4th–6th grade classes [40].

Another unique approach to resilience-building interventions for early childhood educators to address their work stress was implemented by Fulchini (2018) [41]. A biofeedback intervention allows for voluntary control of physiological mechanisms. In the intervention, participants received haptic silent feedback whenever the Spire device detected prolonged tense breathing of the participants for over two minutes [41]. This enables participants to become aware of what triggers their stress in the classroom, observe their thoughts and emotions at that moment, and practice self-compassion through deep breathing.

The MindUP curriculum implemented by Killion (2019) used mindfulness training to improve self-efficacy and perceptions of the school climate [42]. The training module initially had a four-unit, 15-lesson curriculum developed for students, and was modified to a four-unit, 4-lesson curriculum with educators as targeted participants. The four units were entitled Getting Focused, Sharpening Your Senses, All About Attitude, and Taking Action Mindfully. The approach taken throughout the training was a combination of direct instruction, individual and small group activities [42].

Another intervention that implemented a mindfulness approach used the Mindfulness Coach smartphone application that was initially developed and used for PTSD patients [43]. The application can also be used by anyone as a free self-guided mindfulness course, where it will prescribe specific mindfulness meditation practices based on 19 questions completed by users. Participants from this study were principals of K-12 public schools. As the principals hold managerial responsibilities for ECE of children between 5 and 6 years old, this study is included in the systematic review. Participants were randomly assigned into control and treatment groups. The treatment group used the mindfulness application whilst the former group completed only the pre- and post-assessments.

Lang et al. (2020) implemented the Social Emotional Learning for Teachers (SELF-T) online course, which consists of five lessons where participants are taught to reflect on how stress affects their body and how they respond towards stress, and they are introduced to new stress-reduction strategies [44]. It adopts the Learn, Explore, Apply, Demonstrate format that enables the participants to apply and explore the concepts learnt through small exercises, all at their own pace. Initially, 69 participants were recruited from 18 centres of different quality-rated programs, but only 63 completed responses were analysed [44].

Another recent intervention by Hatton-Bowers et al. (2020) also conducted a mindfulness- based intervention on early childhood professionals using an online module [45]. The practice of mindfulness and compassion incorporated in the online module, entitled Mindful Practice for ECE Professionals: Begin the Journey, increases the mental and occupational well-being of individuals. Hatton-Bowers et al. (2020) highlighted how mindfulness may act as a coping resource that promotes resilience and social–emotional competence in ECE settings. Outcomes of the module range from learning about mindful practices, planning their incorporation into daily activities, recognizing their association with effective teaching, to understanding their contribution to stress management [45].

Susman-Stillman et al. (2020) took a different approach in analysing an intervention for ECE professionals by interviewing the reflective supervisors responsible for conducting reflective supervision/consultation (RS/C) sessions [46]. This intervention was based on models of a relationship-based service delivery, where professionals meet regularly with their trained supervisors to reflect on their own emotional reactions and others towards the work challenges faced, and integrate these perspectives with theories and into daily practices [46].

MyTeachingPartner (MTP) program is a professional development intervention used in ECE to promote high-quality teacher–child interactions [47]. Similar to the supervision approach from the RS/C intervention [46], the MTP applies a coaching model where the coaches analyse video-recordings of teachers in their classrooms and provide individualized feedback to the observed teachers based on evidence-based practices on teacher–child interactions. The program provides both emotional and instructional supports to teachers, and has been observed to improve job satisfaction and motivation of teachers [47]. Bayly et al. (2020) analysed data on the outcome of MTP interventions obtained from a longitudinal study conducted on the effectiveness of two professional development interventions for ECE professionals between 2007 and 2011 [48].

Hepburn et al. (2021) conducted a 6-week yoga-based intervention for early career teachers from 49 schools teaching educational levels between prekindergarten and grade 12 [49]. The first two weekly sessions involved topics related to psychological well-being, where the participants were introduced to impacts of stress and breathing techniques for relaxation. The second two weekly sessions highlighted the importance of diet, gut health and exercise, and the relationship between mental health and the digestive system. During the last two weekly sessions, the content of the intervention focused on interpersonal well-being. Throughout the sessions, the contents were delivered using reflection activities and guided meditation exercises. Participants were also given materials as guidelines for performing physical practices at home. Both quantitative and qualitative responses were collected to evaluate the intervention, but only the quantitative data were reported in the paper [49].

In a recent study, Jones et al. (2021) conducted an intervention involving behavioural activation on early career teachers between kindergarten and 12th grade (K-12) [50]. This intervention has been extensively used in treating depression [51,52], as it is a cognitive behavioural therapy skill that focuses on activating positive emotions through behaviour. The intervention addresses the effort–reward imbalance by increasing the number of mastery activities, which then subsequently increases rewards within a person’s environment such as improved self-esteem and happiness. During the first session of the intervention, the trainer assessed the participants and discussed homework assignments related to their goal settings, mood and activity monitoring, action plans and scheduling. In the second session, the trainer reviewed the activities and mood monitored by the participants, and discussed problem-solving strategies to reduce avoidance of the predetermined activation activities. Ratings on the effectiveness and acceptability of the intervention were collected in a follow-up session using a final evaluation questionnaire [50].

Mechelke and Bloomberg (2021) incorporated a personality-based approach using the Enneagram in designing an intervention for teachers [53]. Enneagram is a personality typology that describes personality in nine types, and can explain a person’s behavioural patterns, associated strengths, and challenges. Through self-awareness practices, they can continuously attempt to improve themselves and gain positive social interactions. A one-day Enneagram training session was conducted for teachers with the aim to enhance positive relationships with their students and colleagues. In addition to the training and journal writing encouragement, participants attended one focus-group discussion and two interviews held before and after the intervention [53].

### 3.2. Duration and Frequency of Intervention

The interventions implemented in the 15 studies varied in terms of duration per session and frequency throughout the intervention (as shown in Table 2).

The Personal Growth Program developed by Bokaba (2011) was a three-session intervention based on group discussions [36]. The duration and frequency of meetings of the intervention is unclear. After the formal programme ended, the participants were encouraged to continue meeting up as support groups and were evaluated after one year. Varying responses were reported on the support group meetings, where at most, one group met six times in a period of six months. Participants from groups that did not meet commented that this was due to lack of time, long distance, laziness, and novelty of support groups amongst colleagues.

The exercise program carried out at the two preschools selected by Júníusdóttir (2015) varied in terms of duration, as it depended on the managerial implementation [37]. One preschool allocated 30 min during teachers’ break with the frequency of twice per week, whilst the other preschool allocated 20 min with the same frequency. Both managers were satisfied with the outcome of program, and the implementation was continued even after the study ended.

The reflective supervision program conducted by Lepore (2015), although it was not preceded with a needs assessment, stemmed from the concerns of increased mental health referrals from the first-recruited preschool [38]. The positive responses from the participants resulted in the recruitment of other interested centres. At each centre, participants attended a weekly or biweekly meeting with a duration of 45 to 90 min. Implementation of the intervention was continued even after the study ended.

Christian (2017) collected data from six elementary schools within a large urban school district in the Western region of the USA [39]. The 2–2.5 h duration allocated varied depending on the control and intervention groups, where the former had a 2 h discussion and the latter had a 1–1.5 h of ARC training and a 1 h discussion. Following the intervention, the control group was also offered to participate in the ARC training.

In the three-phase collegial reflection intervention, Nilsson et al. (2017) emphasized limiting it to one meeting only for the first phase and negative evaluation, and continuing the subsequent meetings for the positive evaluation phase to highlight the salutogenic perspective of the chosen issue addressed by participants [40]. A strict duration of 30 min was allocated for the meetings to ensure a short but purposeful discussion, with participants in each group meeting once a week at the school.

The Spire device used in the biofeedback intervention is a wireless tracker clipped on the clothes near the waistline or chest area [41]. Participants were asked to wear the devices during their daily classes for 29 days. Day 1–3 breathing data were used as the pre-test baselines, and Day 26–29 were used as the post-test baselines. The breathing data recorded during the daily classes (over a 6 h duration) were averaged to compute the mean breath per minute and compared between the two baselines.

Killion (2019) fitted the lessons from the MindUP curriculum into weekly sessions for four consecutive weeks [42]. Each session had a duration between 45 and 60 min. Although the intervention ended after four weeks, participants were provided with additional resources after each session to access at their own volition. The curriculum had the advantage of being cost-effective and user-friendly, as it has one manual that is accessible for users and can be easily understood by educators.

No restriction of duration was placed on participants using the Mindfulness Coach application intervention [43]. They were reminded weekly by the app to perform the meditation practices. The practices were assigned based on levels, where participants had to complete each level before moving to the next level. Throughout the intervention, the app tracks usage and records the practice log and duration spent on each meditation session. Coggin (2019) highlighted that the decision of a 4-week intervention instead of the typical 8-week period for mindfulness interventions is to encourage more participation, and it requires less time commitment from the participants.

Participants in the SELF-T intervention conducted by Lang et al. (2020) had a period of two weeks to complete a 3 h online course at their own time [44]. Lessons in the intervention were presented at the Virtual Laboratory School, allowing the participants to apply the knowledge they acquired into practice in a virtual environment.

The online mindful practice module used by Hatton-Bowers et al. (2020) received responses from professionals providing indirect and direct care to children [45]. Focusing the analysis on professionals providing direct care, the study analysed 548 responses from the open-ended question given after the module is completed. The question asks participants to share something they learnt from the online module. Analysis of the qualitative themes identified positive statements from the participants, highlighting valuable information garnered from the module, and their increased intention of sharing with colleagues and promoting training among staff.

The regular meetings conducted in schools of participants interviewed by Susman-Stillman et al. (2020) were one of the strengths of the intervention, as they provided consistent and specific time and space for teachers to reflect on the challenges they faced during work [46]. No restriction was placed on the duration of meeting, but it took place either weekly or monthly, depending on the teachers.

The MTP is a continuous intervention where video-recording sessions and coach observations are conducted over a two-week period [47]. It is unclear how frequently the observations and coaching occurred between the two-week period, but the process was repeated throughout the year.

In the yoga-based intervention conducted on early career teachers, the duration of weekly sessions varied depending on the contents covered during the sessions [49]. Sessions 1–3 ranged between 20 and 45 min, whilst the last three sessions were each conducted for 60 min. A maximum of 20 min practice length was prescribed for the guided meditation and yoga sequence practices at home, whilst the breathing practices were determined by the participants.

The one-to-one sessions conducted in the behavioural activation intervention for ECE professionals were held virtually using the Zoom web conferencing platform [50]. The study implemented a multiple probe design for data collection, where repeated but discontinuous measurements were collected from participants. Prior to the intervention, participants were required to provide baseline responses from chosen questionnaires, collected between three and five data points within a period of one to two weeks. The intervention phase was completed within two weeks, with a 45 min session conducted per week. Between the two sessions, the participants were suggested to spend no more than 30 min each day on their planned activation activities.

In the one-day Enneagram training, the participants were first interviewed for 30 min followed by a 6 h Enneagram training given either in-person or using the WebEx platform to all participants [53]. They were given a notebook and encouraged to write down their daily interactions to instigate self-awareness and self-reflection processes. Each participant attended a one-hour focus group discussion near the end of week two of the intervention. The second interview was conducted after the fourth week of the intervention. Within the four weeks between the training and end of intervention, participants were required to practice the learnt self-awareness skills through reflection practices.

### 3.3. Interventions Based on Needs Assessment

With reference to Table 2, four studies performed a needs assessment prior to the intervention [36,37,39,40]. Bokaba (2011) performed a needs assessment at the early stage of the study to identify challenges and opportunities professionals involved in early childhood development faced [36]. During this assessment, four ECD-trained principals from different preschools agreed to the semi-structured interview. Findings from the assessment contributed to the development of Personal Growth Program which was implemented on 65 teachers in early childhood development. The participants were divided into groups based on schools of five residential areas.

In order to identify preschools that integrated employees’ exercises in their daily work routines, Júníusdóttir (2015) collected online survey responses from 130 preschools [37]. Out of eight preschools that reported integrating such programs, two of these preschools misunderstood and described employees’ physical activities with children. Managers from two of these preschools agreed to be interviewed and gave their feedback on the exercise programs. One preschool has four departments, with children between 10 months and 6 years of age. The second preschool has two departments with children of 18 months to 6 years of age. Both preschools were taking initiatives towards a health-focused culture in their school.

The ACHIEVER module implemented by Christian (2017) was based on a readiness assessment conducted prior to the intervention on large scale, involving a large urban school district [39]. Analysis of the assessment identified barriers that can hinder the implementation of evidence-based practices in school. The participants were nominated by school principals as teachers from kindergarten to 5th grade who were experiencing significant job stress and burnout and could benefit from interventions that could help them in developing healthy lifestyle practices and enhanced well-being.

Similar to [36], Nilsson et al. (2017) also conducted a needs assessment prior to its intervention to identify the challenges faced by teachers in the selected school [40]. During this assessment, 7 teachers from a total of 19 attended focus group interviews and individual interviews to provide feedback on issues they face as teachers. The developed intervention was then implemented on 21 teachers from the same school, ranging from preschool to grade 6 levels.

Although Jones et al. (2021) did not conduct a needs assessment of the centre prior to participant recruitment, the intervention itself is designed to assess the participants individually based on their goals and values to facilitate in planning and scheduling their tasks during the intervention [50]. This is further explained in the following section, where participants were recruited based on their significant levels of stress and emotional exhaustion.

### 3.4. Participant Characteristics

The 15 interventions varied in terms of the number of centres involved and the teaching level of participants. Four studies conducted the intervention in two or fewer schools [37,40,41,53], whereas the other eleven studies recruited participants from at least five different schools. Schools recruited in the interventions reported by Bokaba (2011), Júníusdóttir (2015), Lepore (2015), Lang et al. (2020), and Bayly et al. (2020) were all ECE centres [36,37,38,44,47]. Participants analysed from the online module intervention by Hatton-Bowers et al. (2020) were professionals working in the ECE field with differences in terms of centre, home, school or preschool settings [45]. Similarly, the reflective supervisors recruited by Susman-Stillman et al. (2020) were professionals working within the ECE field from state organizations for infant mental health [46]. The remaining eight studies recruited participants from public schools with classes ranging from preschool to 12 grade, where a fraction of the participants in each study were teaching preschool or kindergarten at the respective schools.

Fulchini (2018) chose one public charter elementary school with low socio-economic status, located in communities of poverty, serving mainly low income and racial or ethnic minority populations [41]. Novice teachers were identified as being within the first five years of teaching in an inclusive (two or more students with disabilities educated with non-disabled peers) classroom and having at least a Bachelor’s degree.

The demography of participants in the intervention reported by Killion (2019) reflects the diversity of teachers, where 8 teachers were teaching preschool, 10 elementary, 1 middle school and 10 taught multiple grades [42]. The job descriptions provided by participants were general education classroom teachers, non-instructional support staff, interventionist, special education teacher, gifted/talented teacher, and teacher leader/integrated arts teacher. Of the 29 participants, their experience in education ranged from 1 to 25 years.

Participants interviewed by Susman-Stillman et al. (2020) were reflective supervisors that were trained to provide RS/C to ECE professionals and were also ECE professionals themselves [46]. They have been on both sides of the RS/C session, as a trainer and trainee. A total of 210 reflective supervisors were recruited using a snowballing strategy from 38 locations of infant mental health organizations.

The two-phase longitudinal study by Pianta and Burchinal (2016) recruited teachers from 200 ECE centres [48]. From the 427 teachers that participated in the first-phase intervention involving coursework on instructional interactions, 95 teachers dropped out and 69 new teachers were recruited. The participating teachers in the second phase were either assigned to the control group (*n* = 196) or the MTP group (*n* = 205). The teachers had varying years of experience in teaching pre-kindergarten classrooms, from less than one to 38 years [47].

Jones et al. (2021) recruited K-12 teachers from public schools located in districts with critical shortages of teacher placements [50]. The teachers were at the early stage of their career, with 1 to 4 years of teaching experience, and self-identified as experiencing high stress and emotional exhaustion but not undergoing any mental health treatment. A total of three participants completed the intervention, each from different schools. Only one participant taught at an elementary school as an exceptional children teacher, whilst the other two were from high school and middle school. Mechelke and Bloomberg (2021) recruited 16 K-12 teachers from one school district [53]. The teachers held different teaching positions, with five being responsible for kindergarten.

### 3.5. Instruments for Resilience, Well-Being, and Work Stress Measures

From the 15 studies reviewed, 6 focused on qualitative analysis in evaluating the outcome of intervention on either resilience, well-being, or work stress levels of the participants [36,37,40,45,46,53]. The responses were collected through interviews or open-ended questions and the themes were analysed. Outcomes of these interventions were related to improvements in psychosocial, mindful and emotional regulation skills.

In the nine studies that utilized quantitative instruments [38,39,41,42,43,44,47,49,50], all researchers used at least one instrument [42] to measure either resilience, well-being, or work stress. Killion (2019) evaluated the outcome of the *MindUP* module intervention based on the changes in sense of self-efficacy, which is a resilience-related measure, and in the perception of school climate by the teachers.

Measures on stress-related changes were used in eight of the studies to evaluate the outcomes of intervention [38,39,41,43,44,47,49,50]. Bayly et al. (2020) measured teacher anxiety as it relates to perceived self-efficacy, work stress and social support [47]. Lang et al. (2020) used the Perceived Stress Scale to measure general stress over the past month [44]. Coggin (2019) measured the level of mindfulness and work stress of principals using the Administrator Stress Index [43]. Christian (2017) collected subjective well-being from teachers, burnout level based on their emotional exhaustion, and sleeping habits gauged based on average hours of nightly sleep over the past week [39]. Lepore (2015) collected information on teaching stress, with a focus on frustration with parents, and self-reported compassion satisfaction and fatigue, to measure professional quality of life [38].

Fulchini (2018) collected the breathing pattern of teachers for 29 days, and burnout level and stress using instruments specific for teachers [41]. The stress level of participants was also monitored using a simple daily stress level scale after their work during the intervention. Two participants had lower breathing patterns at post-measure. Despite the large amount of information collected on breathing rate, daily stress level, and burnout scores of 15 participants, Fulchini (2018) did not perform further inferential analysis on the outcome measures. No patterns could be identified that could be visually observed between the breathing patterns, stress level, and burnout scores of each participant.

In the yoga-based intervention, Hepburn et al. (2021) also took a physiological approach in measuring level of stress using salivary cortisol samples [49]. Paired t-test analyses were conducted on the changes in cortisol level before and after the completion of intervention, and also the changes between the pre- and post-sessions in the later stage of intervention. Cortisol level has been used in various stress-related research as it reflects the psychobiological mechanisms in the brain triggered in response to high stress [54].

The interventions were also assessed for their effects on the professional development of participants. The focus of professional development was mostly on strategies and skills garnered to address stress-inducing events [44], perception towards professional efficacy and school climate [42] and teacher–student interaction [39].

## 4. Key Findings and Proposed Direction

### 4.1. Key Findings of Systematic Review

This section presents the key findings reviewed from the 15 selected studies. Interventions vary in the approach taken to conduct the intervention, duration, and frequency of sessions, with shorter periods observed in interventions implementing an individual approach and longer periods in interventions where participants were involved in group activities. In studies where the participating institutes decided to implement the interventions in their curriculum [36,37,38], the participants were involved in the intervention on a longitudinal basis, made available throughout the school year. There was also a mixed approach of measuring outcomes of the intervention, mostly either through questionnaires or interviews. Studies using questionnaires performed a pre- and post-comparison of the outcome measures, whereas the interviews were used to analyse the themes identified in the responses provided by the participants. In addition, most of the selected studies involved many educational institutes, or teachers with a wide range of classroom levels.

#### 4.1.1. Psychological Assessment as a Main Measure for Well-Being and Mental Health

Conventional assessment tools of stress among early childhood educators used in the literature show that most research relies on self-report instruments. As these assessments are developed for specific psycho-social measures, the use of these instruments to conduct and assess comprehensive resilience-building interventions may require several questionnaires to be answered by participants. This can be observed in the number of items per questionnaire used in the interventions reviewed in Table 1. Excluding the interview approach and instruments with open-ended questions, the number of instruments used in the interventions ranged between one and six questionnaires [38,39,41,42,43,44,45,46,47,49,50]. The 4-point scale instrument used in [45] to measure perceived learning and program usefulness had only two items, whilst the instruments from the other interventions had a total of 20–85 close-ended items. Each of these questionnaires were used to assess different criteria of the participants, such as demographic characteristics, well-being, and learnt skills.

From the 15 studies reviewed, 8 studies reported positive effects of the interventions implemented on the mental health or well-being of participants. Participants were reported to have improved emotions [37,39,44,49,50], level of stress [38,41,43,44,49], behaviour [37,39,49], and mindfulness [37,43,49] towards themselves and their surroundings. Most of the studies reviewed emphasize the effects of intervention on the skills of participants. These skills can be generally categorized as teaching and psychosocial skills. Only two studies incorporated physiological measures in their intervention, using breathing rate [41] and salivary cortisol [49]. Fulchini (2018) reported lower breathing patterns in two out of nine participants analysed, whereas Hepburn et al. (2021) observed significant reductions in the salivary cortisol level analysed from 17 participants. The quantitative data acquired from physiological measures can be utilized to perform various inferential analyses that can allow for generalization towards the population.

#### 4.1.2. Concerns of Response Bias in Self-Report Assessments

Kwon et al. (2019) raised the concern of using the direct contact approach of collecting self-report information on the teacher’s stress or depressive-related problems [11]. In addition to the changes in responses from a day-to-day basis, teachers may be reluctant to reveal sensitive information due concerns surrounding societal stigma and employment. In a study on stress levels among primary and secondary class teachers, the researchers highlighted the limitation of self-report information as the approach is exposed to method bias and provides weak substantive information, and advocated for an objective approach in assessing physiological stress [55]. Studies using qualitative approaches to assess the interventions used semi-structured interviews to identify the responses of participants towards the intervention [36,37,38,40,53]. Although this approach provided detailed information on the responses of participants towards the intervention, it is difficult to gauge its effect before and after intervention and how the outcomes may be generalized to a larger population.

#### 4.1.3. Challenges in the Group-Approach Intervention: Lengthy Period and Attrition

The group approach is more commonly adopted in the interventions as it boasts experience-sharing and includes more participants per session. Several studies observed a high level of attrition rates among participants. Based on the number of participants analysed after each intervention, as shown in Table 2, six studies performed post-intervention analysis on less than half the number of recruited participants [36,37,40,43,46,49]. Bokaba (2011) and Júníusdóttir (2015) conducted post-intervention interviews on six of their participants [36,37], whereas Nilsson et al. (2017) conducted interviews on three of their participants and received 31 completed interviews from their mailed open-ended questions [40]. The survey was submitted on three occasions to all 21 participants, giving a total of 63 (21 participants × 3) possible surveys [40]. Coggin (2019) recruited a large number of participants but was able to analysed 24% of the initial sample [43]. The qualitative analysis performed in [46] received 97 responses from the anonymous online survey distributed to 210 RS/C supervisors, which reflected 46% of the identified population. Similarly, Hepburn et al. (2021) analysed the data from 47% of their recruited participants who completed the six-week yoga intervention with pre-and post-measures [49]. However, the comprehensive approach adopted using qualitative evaluation and quantitative measures of well-being, stress level and salivary cortisol level of the participants, allows the researchers to analyse the significance of effects throughout and after the intervention.

The attrition issues in studies are often due to incomplete participation or missing information. Participants who opted out of the intervention often did so due to work schedules, time constraints and other conflicting commitments [36,43,49]. Bokaba (2011) reported that the novelty of support groups among colleagues as a continuous platform for ECE teachers to address work stress is one of the reasons their follow-up meetings after the intervention received poor attendance [36]. Hepburn et al. (2021) reported that 19 participants withdrew before the first session, and 7 participants withdrew during the intervention [49]. Missing information or responses from participants is also the main problem in most of the studies reviewed [38,40,42,44]. Post-measures involving open mail questions or qualitative responses are often less responded to by participants [38,40]. Technical issues such as loss of data [40] or data which cannot be retrieved [43] also result in the removal of data from further analysis. Coggin (2019) faced problems in retrieving the performance log report of the smartphone application for the post-measure analysis [43].

#### 4.1.4. Balancing between Multi-Centre and Needs-Based Interventions

Findings based on visual observations and qualitative analysis limit generalization towards the intended population. The quantitative approach to analysing findings allows for generalization to a broader population and provides causal evidence for the research questions, whereas qualitative analysis provides diversities in the information being explored [56]. This can be observed in the four studies where needs assessments were conducted through group discussions and semi-structured individual interviews [36,37,39,40]. Bokaba (2011) researched residential areas in South Africa, whereas Nilsson et al. (2017) researched a large urban district in US. Bokaba (2011) and Nilsson et al. (2017) observed the need for social support amongst teachers, but the opportunities and capacity for the centre to provide such interventions are influenced by various factors such as geography and culture [36,40]. Júníusdóttir (2015), on the other hand, used an online survey to identify which preschools integrated exercise activities for employers [37]. Interviews with managers of the two preschools chosen for intervention helped in identifying their plans to implement exercise policies for their employees. There was also an increase in one-to-one approaches coupled with small group activities to deliver the intervention, as shown in more recent studies conducted between 2020 and 2021. This approach allows the activities performed during the intervention to be more personalized and to specifically address the needs, challenges, and strengths of each teacher.

### 4.2. Proposed Direction for Resilience-Building and Work Stress-Management Programs through Physiological Measures

In addressing the challenges of building resilience through group approaches and using psychological assessments, as discussed in Section 4.1, we propose the usage of physiological measures for well-being and mental health to build resilience amongst teachers in ECE settings. The brain is an excellent source of assessing stress, as it is acutely sensitive to the intricacies and detrimental effects of stress on both functional and anatomical levels [57]. This proposed approach is based on the cognitive activation theory of stress, where the stress response is a general alarm in the homeostatic system that produces neurophysiological activation of incremental arousal states [58]. When a person encounters stressful situations, the brain activates cognition in the brain stem and supra-reticular activating system (limbic and frontal lobe) and evokes further chemical changes throughout the body [58].

Amongst the modalities of brain imaging, electroencephalography (EEG) has the advantages of being non-invasive, and having high temporal resolution, low cost, and portability. Stress resilience is a measure of an individual’s vulnerability towards stress and perceiving adverse events as minimally threatening and developing adaptive physiological and psychological responses [59]. Researchers have observed the link between resilience and the prefrontal cortex and amygdala regions [60,61,62]. An increase in prefrontal oxygenation as a response to high mental effort highlights the role of the prefrontal cortex as a crucial brain area [63] in mitigating stress, as it is key for cognition in sustaining coping strategies. Stress susceptibility pathways can be modulated later in life through targeted behavioural intervention, supporting the plasticity mechanism [64]. This has also been suggested through the implementation of neurofeedback training for resilience in both healthy and clinical populations [65].

Furthermore, we also propose the integration of virtual environments to mimic real-life scenarios that reflect the challenges faced by different workplace settings. Various workplace settings have been used to design virtual environments for simulation and training purposes [66,67,68]. VR technology has long been used in the medical field for therapies [69,70,71]. In addition to its flexibility in design and immersive characteristics, the technology has also been recently integrated with physiological monitoring devices to monitor performances of users during the immersive experience [72,73,74]. The approach of an integrated EEG-VR system for a resilience-building intervention can also address the need for multi-centre interventions, as both systems have the advantages of portability and low setup cost. With the rapid advancement of technologies, both devices are also becoming more accessible and user-friendly.

## 5. Pilot Study towards an EEG-VR Resilience-Building Intervention

In an effort towards the proposed direction for resilience-building interventions for early childhood educators, we conducted a pilot study to evaluate the feasibility of monitoring the frontal brain activity of teachers in a virtual classroom. The EEG device is a non-invasive brain-monitoring tool that has been used in various clinical and health science practices, and has the advantage of being portable and having high temporal resolution. It has been used to monitor brain activities in virtual environments to gauge how an individual reacts to a simulated real-life scenario. The use of EEG in resilience-building interventions for teachers in ECE allows for the monitoring of mental health and the effects of the intervention on resilience levels, whereas VR technology allows the intervention to be designed based on the needs and actual settings of the centre itself. Hence, in this pilot study, we monitored the frontal brain activity of teachers’ during a mental task in a virtual classroom and identified its correlation to their resilience scores.

The study was conducted on 25 teachers recruited from the National Child Development Research Centre (NCDRC) in Universiti Pendidikan Sultan Idris (UPSI). The participants were healthy working adults involved in the ECE at NCDRC, aged 18 or above, and had normal or corrected-to-normal vision. The participants were screened as healthy by answering “No” to questions on having a diagnosis of hypertension without intake of antihypertensive medication, positive drug anamnesis, history of psychiatric diseases requiring inpatient treatment longer than 2 weeks, neurological disorders, and malignant disease. The ethical approval for this study was obtained from the UPSI Ethics Committee (2020-0121-01), and written informed consent was obtained from all participants prior to the experiment. The CD-RISC questionnaire was used to measure the resilience scores of each participant [75].

### 5.1. Setup of Virtual Classroom

**Figure 2:**
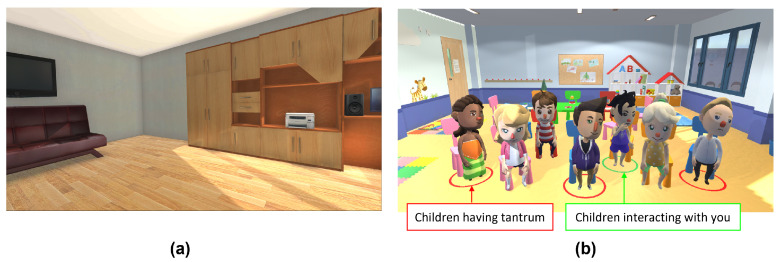
View of virtual environment for: (**a**) Enclosed room during resting with eyes open; (**b**) Classroom during mental task.

The experiment involved using the Oculus Quest headset to display the virtual environment of an enclosed room and a classroom (as shown in Figure 2) to the participants. The MITSAR-EEG-202-31 device was used to record the EEG signals from the participants. Each participant was required to complete four sessions of EEG recording, with the following flow:

1. 2 minutes of resting condition in a virtual enclosed room;
2. 4 minutes of mental task condition in a virtual classroom;
3. 2 minutes of resting condition in a virtual enclosed room;
4. 4 minutes of mental task condition in a virtual classroom.

During the resting condition, the participants are shown a view of them sitting on a sofa in a virtual enclosed room. In the virtual classroom, the participants are presented with children interacting with them as their teacher. The participants are given a task during the virtual class to use mental strategies such as imagination or calming thoughts to eliminate the red circles around the children in the classroom. The red circles indicate a child exhibiting tantrum behaviour.

### 5.2. EEG Recording and Processing

The EEG signal is usually characterized into five brainwaves defined by specific frequency ranges that reflect different mental states of an adult brain: delta (0.3–4 Hz, deep sleep), theta (4–8 Hz, drowsy), alpha (8–13 Hz, relaxed), beta (13–30 Hz, focused) and gamma (30–100 Hz, higher mental activity). Alpha brainwaves in the frontal area of the brain have been studied extensively in stress-related literature [76], where alpha asymmetry index favouring the right frontal area (i.e., greater left frontal activity) is associated with approach strategies, whilst a higher alpha asymmetry index in left frontal area is associated with withdrawal/avoidance strategies [77,78,79,80]. In this pilot study, the alpha asymmetry index is calculated for both resting and task conditions in order to identify its correlation with the resilience scores of childcare teachers.

The EEG signals are recorded using a 32-channel EEG cap at a sampling rate of 500 Hz. Six electrode channels that represent the frontal area of the brain are chosen (i.e., Fp1, Fp2, F7, F3, F4 and F8) to compute the alpha power for both resting and task conditions, using the following processing steps. From the six electrodes, three pairs of alpha asymmetry indices are computed for Fp2-Fp1 (prefrontal), F4-F3 (mid-frontal) and F8-F7 (lateral frontal).

1. Band-pass filtering between 1 and 45 Hz EEG signals to remove power-line noise;
2. Automated artifact rejection using the HAPPE pipeline [81] and MARA toolbox [82,83];
3. Reference-free quantification using CSD toolbox [84,85,86];
4. Computation of relative alpha power [87] for both resting and task conditions.
5. Computation of alpha asymmetry index using log-transformed alpha-power density values [79,80]
  (1) $$Alpha\;symmetrysymmetry\;index=\ln\left(\frac{Alpha\;{power}_{right}}{Alpha\;{power}_{left}}\right)$$

### 5.3. Statistical Analysis

The relative power values for each session are tested using the Shapiro–Wilk and Levene tests for normality and equality of variances. To observe the difference in power values between right and left frontal areas, an independent t-test is used for parametric testing, and a Mann-Whitney U test is used for non-parametric testing, to compare the relative alpha power values of all three pairs of electrodes. Correlation analysis is performed using Spearman’s rank-order rho to identify the association between CD-RISC resilience scores and the three alpha asymmetry indices in both resting and task conditions.

### 5.4. Preliminary Findings

A total of 25 teachers amongst the childcare professionals working in the NCDRC, UPSI, passed the health screening assessment and were recruited as participants for this study. The participants were healthy adults with no neurological complications with a mean age of 33.52 ± 5.5 years old. Demographic characteristics of participants are presented in Table 3.

The CD-RISC scores ranged from 53 to 98.9 (median = 68, IQR = 13.5). Following the CD-RISC manual guide, the CD-RISC scores can be described using quartile statistics to divide the participants into four groups from lowest to highest resilience level [88]. In this study, the range of scores and number of participants in each quartile were as follows: n_Q1_ = 6 (i.e., least resilient, score 53–60.75); n_Q2_ = 7 (score 60.75–68); n_Q3_ = 6 (score 60.75–68); n_Q4_ = 5 (i.e., most resilient, score 74.25–87). One resilience score of the male participants was observed to be an outlier, with a score of 98.9.

**Figure 3:**
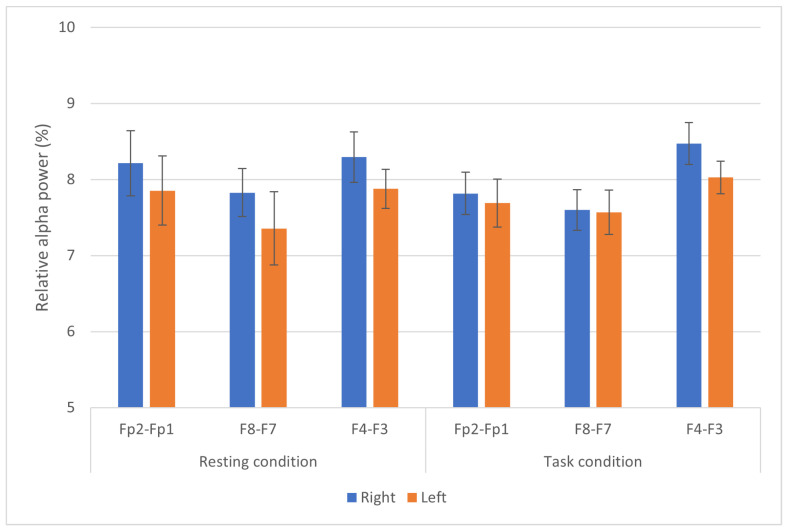
Relative alpha power (%) between the respective right (Fp2, F8, F4) and left (Fp1, F7, F3) brain areas. No differences were observed at all three electrode pairs during both conditions.

Relative power of the alpha brainwaves throughout the experiment is calculated for each session, and grand averaged into resting and task conditions. Figure 3 presents the relative alpha power (%) at the prefrontal (Fp2-Fp1), lateral frontal (F8-F7) and mid-frontal (F4-F3) areas. Comparison analysis of the relative power of alpha brainwaves between the right and left frontal brain areas indicated non-significant differences (*p* > 0.05) between the hemispheres at all three electrode pairs.

**Figure 4:**
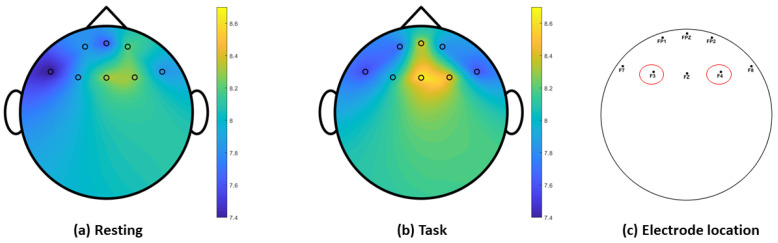
Brain topography of relative alpha power during (**a**) resting and (**b**) task conditions, and (**c**) location of electrode pair with alpha asymmetry index correlated with resilience scores.

Brain topography of the grand-averaged relative alpha power in Figure 4 illustrates high alpha activity in the F4 electrode location during the task condition, but this is less apparent during the resting condition. The alpha asymmetry index quantifies the differences between the right and left frontal brain activities.

**Figure 5:**
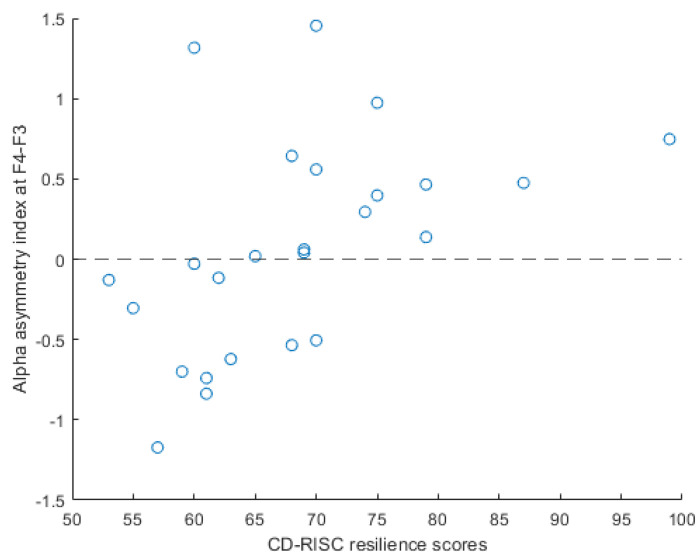
Relationship between CD-RISC scores and the averaged alpha asymmetry index during task conditions (task_averaged_: rho = 0.49, *p* = 0.01). Participants with lower scores are observed to have negative alpha asymmetry index at F4-F3.

Correlation analysis between the alpha asymmetry indices of all three electrode pairs and resilience scores indicated a positive correlation at the F4-F3 electrode location (as shown in Figure 4) during both task sessions (task_1_: rho = 0.38, *p* = 0.06; task_2_: rho = 0.40, *p* = 0.05). This correlation is illustrated in the scatter plot between the resilience score of each participant and their respective alpha asymmetry index at F4-F3, averaged from the two task sessions (task_averaged_: rho = 0.49, *p* = 0.01) in Figure 5, where participants with lower resilience scores had a negative alpha asymmetry index. During the resting sessions, weak correlation is observed between the alpha asymmetry indices and the CD-RISC scores (rest_1_: rho = −0.02, *p* = 0.90; rest_2_: rho = 0.11, *p* = 0.61).

Alpha brainwaves reflect the resting state and are inverse to the cortical activities at the brain area monitored [79,89]. A high level of alpha brainwaves in the right frontal area indicates stronger cortical activity in the left frontal area, which has been associated with tendencies towards approach strategies [77,78,79] and tenderness [90]. Aside from being correlated with various brain functions [78,79], frontal alpha asymmetry has also been used in EEG-neurofeedback therapies [91,92]. Findings from this pilot study show that the parameters extracted from EEG signals may be used as physiological measures to monitor changes and improvements in a resilience-building interventions for teachers.

## 6. Conclusions

This systematic review provides an overall view of the implementation of resilience-building interventions among early childhood educators to improve their mental health and well-being. The group approach is more commonly practised in the interventions, along with psychological assessments to measure mental health or well-being. The main challenges of the group approach are its lengthy period and simultaneous involvement of educators during meetings, which may affect the teaching workload of colleagues. Differences in time availability and level of motivation to attend the meetings are also factors that contribute to the success of intervention. There is also an equal need for interventions that address centre-focused issues and ones that can be implemented at multiple centres with similar population demographics. To address these challenges, we propose an integrated EEG-VR approach to build resilience in ECE teachers. The use of VR allows the training to be designed based on the needs of the respective ECE institutes, whereas the monitoring of brain activity using the EEG provides a more personalized delivery of the intervention for the teachers. These advantages can be observed in the preliminary findings, where the frontal brain activity reflected in alpha asymmetry indices correlated with the resilience level of teachers when performing mental tasks but not during resting. By incorporating other endogenous and exogenous factors of individuals, this approach allows the resilience-building intervention to be planned based on the strengths and limitations of the teachers.

## Figures and Tables

**Table 1:** Overview of interventions conducted by selected studies.

Approach	Study Location	Participants	Program	Instruments and Measures	Outcome of Intervention
Group, with facilitator [36]	Schools with Head Start program in 5 residential areas	ECE teachers	Personal Growth Programme designed by identified needs	Group meeting reports per session Semi-structured interview	Self-exploration session—remember positive experiences in life, feelings of empowerment, shared similar experience but handled in unique ways Self-reflection session—self-disclosure depends on who you share with, understanding the importance of acceptance and respect for adults and children Circle of Friends session—importance of sharing with trusted ones and honesty, a very effective program, which starts a process of interaction between participants
Group [37]	Two preschools integrating employee exercise in work routine	Voluntary employees	Exercise program, 20 min group outdoor activity	Online survey—*Employee integrated exercise program availability* Semi-structured interview using phone call—*Implementation and effects of program*	Program improved motivation in work culture, internal morale, fellowship, health conscious, reduced absenteeism, renewal, and reenergized feeling Increased voluntary participation among employees throughout intervention
Group [38]	Five early childhood care centres serving homeless or low-income families.	Experienced (>2 years of RS) and new (<1 year RS) teachers	Reflective supervision	6-item Index of Teaching Stress and Frustration with parents scale—*Work stress* 30-item Professional Quality of Life: Compassion Satisfaction and Fatigue, version 5—*Work stress* Qualitative interview—*Feedback on intervention* 10-item Adverse Childhood Experience—*Childhood experience* 25-item Parental Bonding Instrument—*Teacher and own parents relationship*	Compared to novice group, experienced group had: Similar or decreased frustration levels with parent over the school year, despite having less-optimal parenting background Lower levels of work stress
Group [39]	Six elementary schools in a large urban district	Teachers between kindergarten and 5th grade	ACHIEVER Resilience Curriculum	8-item Teacher Subjective Well-being Questionnaire— *Resilience and well-being* 1 item on sleep habits—*Healthy habits* 3-item Maslach Burnout Inventory, Emotional Exhaustion subscale—*Stress effect* 2-item Teacher–Student Interaction Rating—*Teacher-student interaction* 6-item adapted Usage Rating Profile Intervention—*Acceptability and feasibility*	Compared to control group, intervention group significantly improved in: School connectedness, teaching efficacy and overall well-being Average hours of nightly sleep Emotional exhaustion
Group [40]	One school from preschool to sixth grade classes	Teachers from preschool to sixth grade	Collegial reflection	Digital recordings of reflection meeting—*Social support discussion* Open mail questions—*Feedback on intervention* Individual interviews—*Feedback on intervention*	Paradoxes found at the group level on design of intervention: wanting to decide or be guided, meeting as professionals or persons, looking for safe or new experience Teacher’s experiencing new perspectives and ways of thinking; orientated towards development; question taken-for-granted; common goal and consensus Teachers’ giving and receiving support; sharing thoughts, ideas and experiences; vulnerability; acknowledging and supporting; collegial community
Individual [41]	One low-SES public elementary school	Novice elementary teachers, within 5 years in-service	Breathing Biofeedback; 29 days of wearing Spire device	Spire Biofeedback Breathing—*Breathing pattern* 22-item Maslach Burnout Inventory—Educators Survey—*Work stress effect* 49-item Teacher Stress Inventory—*Work stress* Daily stress level scale—*Work stress* 7–12 min informal final interview—*Social validity*	At post-measure, two participants had significantly lower breathing patterns 78% of participants had reduced perceived levels of stress
Mixed [42]	5 public schools, urban district	Teachers between preschool and 5th grade	MindUP curriculum, modified version	24-item Teacher’s Sense of Efficacy Scale—*Resilience* 54-item Yale School Climate Survey, school staff version, revised edition—*School condition*	At post-measure, participants significantly improved in: Sense of self-efficacy in instructional strategies School–parent community relations
Individual [43]	K-12 public schools across 20 regions in Texas	School principals	Mindfulness Coach smartphone application, self-guided	14-item Freiburg Mindfulness Inventory—*well-being* 35-item Administrator Stress Index—*Work stress*	Non-significant changes in group between pre- and post-measure Increased 8% of mindfulness Decreased 5% of overall administrative stress
Individual [44]	18 centre-based childcare programs	Early childhood teachers	SELF-T, Self-paced online course	12-item newly developed scale on understanding stress and stress-reduction techniques—*Knowledge on stress and stress reduction* 6 items on use of stress-prevention strategies at work—*Knowledge of stress management* 10-item Perceived Stress Scale—*Stress* 10-item Emotion Regulation Questionnaire on positive (reappraisal) and negative (suppression) strategies—*Resilience* 21-item Coping with Children’s Negative Emotions Scale—*Resilience* 11-item Coping with Children’s Challenging Social Interaction Scale—*Resilience* Post-intervention feedback and open-ended questions	At post-measure, participants significantly improved in: Knowledge of stress and its consequences Knowledge of stress-reduction techniques Actual use of stress-reduction strategies Use of reappraisal emotion-regulation strategies Personal perceived stress Expressive encouragement to children Negative emotions Negative social guidance Negative reactions to children, or negative emotion
Individual [45]	Four program types: Centre, Home, School-aged, Prekindergarten/Preschools	Early childhood professionals; direct- and indirect care	2 h Online module: Mindful Practice for ECE Professionals: Begin the Journey; Online module	Two 4-point scale questions—*Perceived learning and program usefulness* One open-ended question	Module received 89% positive ratings on perceived learning and 94% positive ratings on usefulness of information with children and families. Seven themes identified under three broad categories: mindfulness strategies outside early care and education setting, mindfulness strategies inside early care and education setting, and benefits of practicing mindfulness.
Mixed, with facilitator [46]	38 state organizations for infant mental health	Reflective supervisors for ECE professionals	Reflective supervision/consultation	Online survey with 38 questions, including 4 open-ended questions—*Demographics, training in becoming provider, perception of intervention effects*	Themes observed on the effects of intervention: Cultivated emotional skills Increased reflective skills Gained supportive relationships Improved stress management
One-to-one, with facilitator [47]	200 ECE centres	Preschool teachers	MyTeachingPartner, video and coaching	10-item State-Trait Anxiety Inventory—*Teacher’s anxiety* 3 subscales from Teachers’ Sense of Self-Efficacy Scale Short Form (12 item*)—Self-efficacy* 3 subscales from Teacher Stress Index (16 item)—*Work Stress and need for autonomy* 2 subscales from School Climate Index (10 item)—*School climate* CLASS Pre-K observational scores—*Teacher–child interactions*	Three subgroups of teachers identified based on needs of competence, autonomy and relatedness Improved emotional interaction with children similarly in all three identified subgroups of teachers. Improved instructional interaction with children, but more effective in teachers with more confidence and more supported.
Mixed [49]	49 schools with prekindergarten to grade 12 levels	Teachers with 1–5 years in service	Yoga-based intervention; home practice and group practice	15-item Mindful Attention Awareness Scale—*Mindfulness* 8-items Personal Well-being Index—*well-being* 10-item Perceived Stress Scale—*Stress* 22-item Maslach Burnout Inventory Educators Survey—*Work stress effect* 30-item Job-related Affected Well-being Scale—*Affective responses* Salivary cortisol level—*Stress response* Descriptive and subjective questions—*Qualitative evaluation*	Comparing before and after the intervention: Significant decrease in waking and resting salivary cortisol level between before and after intervention Significant decrease in salivary cortisol level between before and after weekly sessions. Significant improvement in perceived stress, attention awareness and subjective well-being Non-significant improvement in positive and negative emotions, emotional exhausiton and depersonalization. Non-significant decrease in personal accomplishment
One-to-one, with facilitator [50]	Public schools in areas with shortages of teacher placements	High-stress and emotionally exhausted teachers	Behavioural Activation Intervention, virtual meetings on Zoom	4-item Perceived Stress Scale—Short Form—*Stress* 9-item Behavioural Activation Depression Scale—Short Form—*Behavioural activation, goal-directed behaviours, social-emotional health* 2-scale, 10-item Positive and Negative Affect Schedule—*Positive emotions* 15-item Final Evaluation Questionnaire—*Effectiveness and acceptability*	Throughout intervention: Only one participant (i.e., elementary teacher) had a consistent increase in well-being, and one had no effect Two participants had consistent increases in positive emotion levels, and one had no effect (i.e., middle school) Poor consistency in decreased perceived stress in all three participants Two participants gave high ratings on relevance, effectiveness and acceptability, and one gave a poor rating (i.e., middle school)
Mixed, with facilitator [53]	One private school with K-12 grade	Teachers with different expertise and >1 year experience (5 kindergarten)	One-day Enneagram training, with journaling and focus group discussion	Daily journal writing—*Social interaction and self-awareness* 11 open-ended questions—Interview 1 9 open-ended questions—Interview 2	Qualitative analysis on practicing learnt: Self-awareness skills: all perceived positive impacts on student (75%) and colleague (all) relationships Social awareness skills: all perceived positive impacts on student (50%) and colleague (all) relationships Mindfulness: 25% perceived positive impacts on student relationships; 25% started utilizing mindfulness Stress management activities: 25% perceived positive impacts on student relationships; 75% changed their practices

Note: ECE—early childhood education; RS—reflective supervision; SES—socioeconomic status; K-12—kindergarten to 12th grade; SELF-T—Social Emotional Learning for Teachers.

**Table 2:** Duration and frequency of intervention.

Duration per Session	Frequency of Session	Period of Intervention	Conducted Needs Assessment	Recruited Participant	Analysed Participant
Unclear [36]	3 sessions	Continuous	Yes	65	6 interviewed
20–30 min [37]	Two days per week	Continuous	Yes	Varied	6 interviewed
45–90 mins [38]	Weekly or biweekly	Continuous	No	37	37
2–2.5 h [39]	Weekly	8 weeks	Yes	67	67
30 min [40]	Weekly (14 meetings)	4 months	Yes	21	3 interviews + 31 returned open-mail questions)
6 h [41]	Daily	29 days	No	13	9
45–60 min [42]	Weekly	4 weeks	No	35	29
No restriction [43]	Daily	4 weeks	No	224	53
No restriction [44]	3 h to complete	2 weeks	No	63	63 (32 returned activity packet feedback)
No restriction [45]	2 h to complete	—	No	680, direct	548
No restriction, but consistent [46]	Weekly or monthly	Continuous	No	210	97
Unclear [47]	Unclear	Continuous	No	427+69	401
Varied between sessions [49]	Weekly	6 Weeks	No	51	24 (17 with salivary data)
45 + 30 min [50]	Weekly + Daily	2 weeks	Yes	6	3
No restriction [53]	Daily + 6 h training	4 weeks	No	16	16

**Table 3:** Demographic characteristic of participants.

Characteristics (N = 25)		Frequency
Gender	Females	24
	Male	1
Age	18–24	2
	25–34	13
	35–49	9
	50–64	1
Marital Status	Single	4
	Married	20
	Divorced	1
Level of education	High school	6
	College or Pre-university	3
	Diploma or Bachelor’s degree	9
	Master’s degree	7
Length of working experience	<1 year	2
	1–5 years	4
	5–10 years	11
	10–15 years	5
	>15 years	3

## Data Availability

The data presented in this study are available on request from the corresponding author.
